# Designing eligibility and evaluation criteria for antibacterial pull incentives: a comparative review of implemented and emerging initiatives

**DOI:** 10.1016/j.lanepe.2026.101849

**Published:** 2026-09-04

**Authors:** Michael Anderson, Emily O'Neill, Gunnar Ljungqvist, Avi Cherla, Zilke Claessens, Elise Schoefs, Nick Crabb, Warren Cowell, Jennifer Cohn, Deepali Patel, Elias Mossialos

**Affiliations:** a Centre for Primary Care & Health Services Research, The University of Manchester, Manchester, UK; b LSE Health, London School of Economics and Political Science, London, UK; c Department of Pharmaceutical and Pharmacological Sciences, KU Leuven, Leuven, Belgium; d Research Foundation – Flanders (FWO), Brussels, Belgium; e National Institute for Health and Care Excellence, Manchester, UK; f Shionogi, London, UK; g Division of Infectious Diseases, Perelman School of Medicine at the University of Pennsylvania, Philadelphia, PA, United States; h AMR Action Fund, Basel, Switzerland

**Keywords:** Antibiotic resistance, Antimicrobial resistance, Research and development, Reimbursement reforms, Pharmaceutical reform

## Abstract

**Background:**

Pull incentives aim to improve the commercial viability of antibacterials, but the criteria determining which products and companies qualify have not been compared across countries. We compared product and company eligibility and evaluation criteria across these incentives.

**Methods:**

We conducted an umbrella review of MEDLINE and Embase for English-language reviews of pull incentives targeting antibacterial innovation and access (2014 to 14 October 2024), supplemented by grey literature searches and the Global AMR R&D Hub dashboard (December 2025). Two reviewers independently extracted criteria from official documentation, using a framework of six product domains (high-priority medical need, relative effectiveness, unmet clinical need, innovative characteristics, health-system impact, and other) and five company domains (antibacterial sustainability, patient access, environmental health, economic criteria, and other).

**Findings:**

We identified 28 pull incentives: 20 implemented (UK, Sweden, Germany, France, Italy, US, Japan, and EU) and 8 proposed or in development (US, Japan, Canada, Australia, Switzerland, and EU). As mechanisms overlapped within countries, the 20 implemented incentives were analysed as nine groups. All nine targeted high-priority medical need and seven (78%) included unmet clinical need. Relative effectiveness featured in six (67%), economic criteria in five (56%), and innovative characteristics, health-system impact, patient access, and antibacterial sustainability in four each (44%); environmental health in two (22%). The UK Subscription Model applied all 11 domains and Sweden's Annual Revenue Guarantee eight (73%). Priority pathogen lists were widely referenced but varied in breadth.

**Interpretation:**

Eligibility and evaluation criteria vary substantially across countries in scope and specificity. Company-level obligations on stewardship, access, and environmental safeguards are applied inconsistently, and most comprehensive in the UK and Swedish models. Greater international alignment around a shared core of criteria, while retaining flexibility for national context and operational feasibility, could reduce uncertainty for developers and strengthen the global incentive ecosystem.

**Funding:**

World Health Organization Regional Office for Europe.

**Research in context**

**Evidence before this study**

We searched MEDLINE and Embase from 2014 to 14 October 2024 for English-language reviews profiling pull incentives targeting antibacterial innovation and access, using terms combining (“antibacterial∗” OR “antimicrobial∗” OR “antibiotic∗” OR “antifungal∗” OR “anti-infective∗”) AND (“incentive∗” OR “policy” OR “policies” OR “reimbursement” OR “regulation” OR “fund∗” OR “contract∗” OR “invest∗”) AND (“research” OR “development” OR “access” OR “clinical trial∗” OR “preclinical trial∗”). We supplemented this with a grey literature search of Google Scholar and government websites, and reviewed the Global AMR R&D Hub dashboard in December 2025 to identify more recent incentives. We identified 29 reviews of incentives targeting antibacterial innovation and access. Most included reviews were narrative and were not formally appraised for quality. Reviews focused on mapping incentives and their mechanisms, and we found no study that has systematically compared the product and company eligibility and evaluation criteria that determine which antibacterials qualify for these incentives, or examined alignment across countries.

**Added value of this study**

We identified 28 pull incentives: 20 implemented in the UK, Sweden, Germany, France, Italy, the US, Japan, and the EU, and 8 proposed or in development in the US, Japan, Canada, Australia, Switzerland, and the EU. We mapped their criteria against six product domains (targeting high-priority medical need, relative effectiveness, unmet clinical need, innovative characteristics, health-system impact, other) and five company domains (antibacterial sustainability, patient access, environmental health, economic criteria, and other). All nine groups of implemented incentives targeted high-priority medical need, but company-level obligations such as stewardship, patient access, and environmental safeguards were applied narrowly and inconsistently, and were most comprehensively integrated in the UK and Swedish models. We also compared the pathogens targeted by each incentive against WHO and national priority pathogen lists.

**Implications of all the available evidence**

The marked variation in eligibility and evaluation criteria across antibacterial pull incentives risks sending inconsistent signals to developers and fragmenting the incentive landscape internationally. Greater alignment around a shared core of criteria, while retaining flexibility for national context, could strengthen the global antibacterial incentive ecosystem and support more efficient, equitable, and sustainable antibacterial development. Multilateral platforms such as the Global AMR R&D Hub, G7, and G20 could facilitate coordinated approaches to incentive design, building on existing frameworks such as CARB-X's stewardship and access standards. Policymakers should weigh the trade-off between comprehensive, multi-domain criteria and operational feasibility, particularly for smaller developers. One option is to distinguish a small set of essential criteria that apply universally, such as targeting priority pathogens and basic stewardship requirements, from supplementary criteria that countries adopt as regulatory and monitoring capacity allows.

## Introduction

In recent decades, few new antibacterials have been developed, with most lacking novel features,^1^ making them vulnerable to the development of cross-resistance with pre-existing antibacterials. In 2022, the World Health Organization (WHO) described the current antibacterial pipeline as insufficient to combat rising antimicrobial resistance (AMR).^2^ Scientific, economic, structural, and regulatory ‘barriers’ continue to limit the development of novel antibacterials.^3^ Over the last decade, investment has increased in incentives to stimulate antibacterial research and development (R&D) and novel antibacterials' commercial viability.^3^ Push incentives (e.g. direct funding and grants) can reduce the cost of R&D and have improved the quality of the basic science and preclinical pipeline^4^; pull incentives (e.g. financial rewards linked to R&D success, reimbursement reforms and regulatory changes) can increase revenues from new and existing antibacterials and encourage R&D towards the end of the clinical pipeline.

International alignment of eligibility and evaluation criteria is particularly important for pull incentives that target both innovation and sustainable access. Uncertainty about what criteria must be fulfilled in order to obtain rewards discourages companies from investing in antibacterial R&D.^5^ By pooling commitments and harmonising approaches, countries can provide developers with a stable return on investment while supporting equitable global access to antibacterials.^6^ At the same time, some flexibility in eligibility and evaluation criteria will remain necessary to reflect differing national public health priorities, resistance patterns, health-system contexts, and the specific objectives of individual pull mechanisms. Effective international alignment should therefore focus on shared core principles and minimum standards, while allowing countries to adapt implementation to local contexts. Criteria may also vary according to whether the primary objective of the pull incentive is to promote novel antibacterial R&D or to secure sustainable access to existing antibacterials. However, in practice, pull incentives often state both objectives.

The objective of this paper is to identify product and company eligibility and evaluation criteria for pull incentives that aim to stimulate investment in R&D, launch, equitable access, and market sustainability of antibacterials (hereafter referred to as antibacterial innovation and access). We focus on ‘pull’ rather than ‘push’ incentives because international alignment of eligibility and evaluation criteria is potentially more relevant for pull mechanisms, which aim to improve market conditions for late-stage antibacterial developers, and reinforce stewardship and access commitments.

We first review the criteria used in current and proposed pull incentives to signal priorities to industry. We then examine target pathogens and product profiles to assess alignment with global health needs. Finally, we present country case studies to illustrate how the incentives are implemented and to identify opportunities for better international coordination.

## Methods

Our process was divided into two stages. First, identification of implemented or proposed pull incentives; second, identification and categorisation of product and company eligibility and evaluation criteria for each incentive. We focused on small-molecule antibacterials, and excluded incentives for antibodies, antivirals, or antifungals.

### Identification of relevant pull incentives

We conducted a literature review following Joanna Briggs Institute guidelines,^7^ including any English-language literature review published between 2014 and 2024 that profiled examples of pull incentives targeting antibacterial innovation and access. We also conducted a grey literature review using Google Scholar (first 200 hits),^8^ and government websites. To ensure we identified recent pull incentives implemented as of 2025, we reviewed the Global AMR R&D Hub dashboard in December 2025.^9^

We had no exclusion criteria related to review type and therefore included narrative reviews in our analysis. A broad range of search terms was applied to titles and abstracts contained within two databases (Medline and EMBASE) up to 14 October 2024 (See Appendix Table S1). MEDLINE and Embase are two of the most widely used biomedical databases because of their reliability and comprehensive coverage,^10^ and a recent study has indicated that two databases are sufficient to minimise the risk of missing relevant studies.^11^

Two reviewers independently screened titles and abstracts with 25% overlap for reliability assessment. Cohen's kappa was calculated to assess inter-rater agreement (99.2% agreement, and 0.78 Cohen's Kappa). Discrepancies were resolved through discussion. Once reviews were identified, all pull incentives targeting antibacterial innovation and access were extracted. Incentives were categorised as follows: (1) relevant country or region; (2) mechanism type; (3) whether implemented or proposed; (4) number of years operational; and (5) whether antibacterial-specific or not.

### Identification of product and company eligibility and evaluation criteria

Two reviewers independently extracted detailed eligibility and evaluation criteria from the official websites, policy documents, and technical guidance associated with each pull incentive (ZC and MA). We developed a structured extraction framework based on Anderson et al., 2023,^3^ who based their approach on Simpkin et al., 2017.^12^ This framework categorised the criteria into six product domains (targeting high-priority medical need, relative effectiveness, unmet clinical need, innovative characteristics, health-system impact, and other) and five company domains (antibacterial sustainability, patient access, environmental health, economic criteria, other).

The extraction framework was pilot-tested using criteria from the UK subscription model and German reimbursement reforms, then iteratively refined. Product criteria encompassed antibacterial characteristics, e.g. targeting priority pathogens, novel mechanisms of action, or new chemical classes. Company criteria captured terms and conditions for manufacturer eligibility, including environmental standards, stewardship commitments, and supply security (Table 1). Extracted criteria were synthesised narratively for each incentive and comparatively across countries to identify patterns and gaps.

**Table 1:** Data extraction criteria.

Product eligibility and evaluation criteria	Potential examples
Targeting high-priority medical need	The product must: • Target priority pathogens • Target life-threatening or chronically debilitating disease
Relative effectiveness	• Effectiveness compared to current standard of care (from NICE subscription model)
Unmet clinical need	• Activity against clinical indications with unmet needs in different populations (i.e. no or limited clinically equivalent therapeutic options available) (from UK subscription model)
Innovative characteristics	• No cross-resistance • New target • New mode of action • New chemical class/entity (based on WHO pipeline classification of innovative characteristics)
Health-system impact	• Risk of adverse events and drug–drug interactions • Costs and implications for different mode/route of administration • Dose frequency • Product stability and storage • Monitoring of treatment (from NICE subscription model)
Other product characteristics	• Penetration to relevant anatomical sites of infection • Reduced impact on microbiota • Any other relevant product eligibility and evaluation criteria (from NICE subscription model)
**Company eligibility and evaluation criteria**	**For example, the company must:**

Antibacterial sustainability	• include stewardship programmes and/or approval by specialist infectious disease physician prior to prescription • limit promotion activities to information/education rather than encouraging overuse
Patient access	• facilitate consistent access across different markets including to low- and middle-income countries (LMICs) • ensure supply security, e.g. diversification of manufacturing capacity or technology transfer agreements
Environmental health	Comply with: • antibacterial manufacturing standards to reduce the development of resistance and ecotoxicity in the environment • independent assessment of manufacturing standards
Economic criteria	Declare: • Company size (small and medium enterprises (SMEs) may be ineligible) • Whether non-profit or for-profit • Whether it accepts dilutive or non-dilutive funding
Other	• Meet relevant social value requirements, such as contributing to net zero carbon emissions • Have sound employment conditions and processes • Any other relevant company eligibility or evaluation criteria

The product and company eligibility and evaluation criteria were collated for each pull incentive, and summarised narratively by country. Data collection took place between January and March 2025, and was updated in December 2025 to reflect recent developments. Several countries operated multiple schemes. We were unable to extract comprehensive eligibility and evaluation criteria for pull incentives not yet implemented as their criteria were not publicly available and/or remained subject to change. Therefore, we divided our narrative synthesis into ‘implemented’ versus ‘proposed or in-development’ initiatives.

### Role of funding source

The World Health Organisation Regional Office for Europe provided financial support for this work, and hosted meetings of the Novel Medicines Platform antibacterial subgroup. They did not influence study design, data collection, data analysis, data interpretation, or writing of the report.

## Results

### Pull incentives identified

**Fig. 1:**
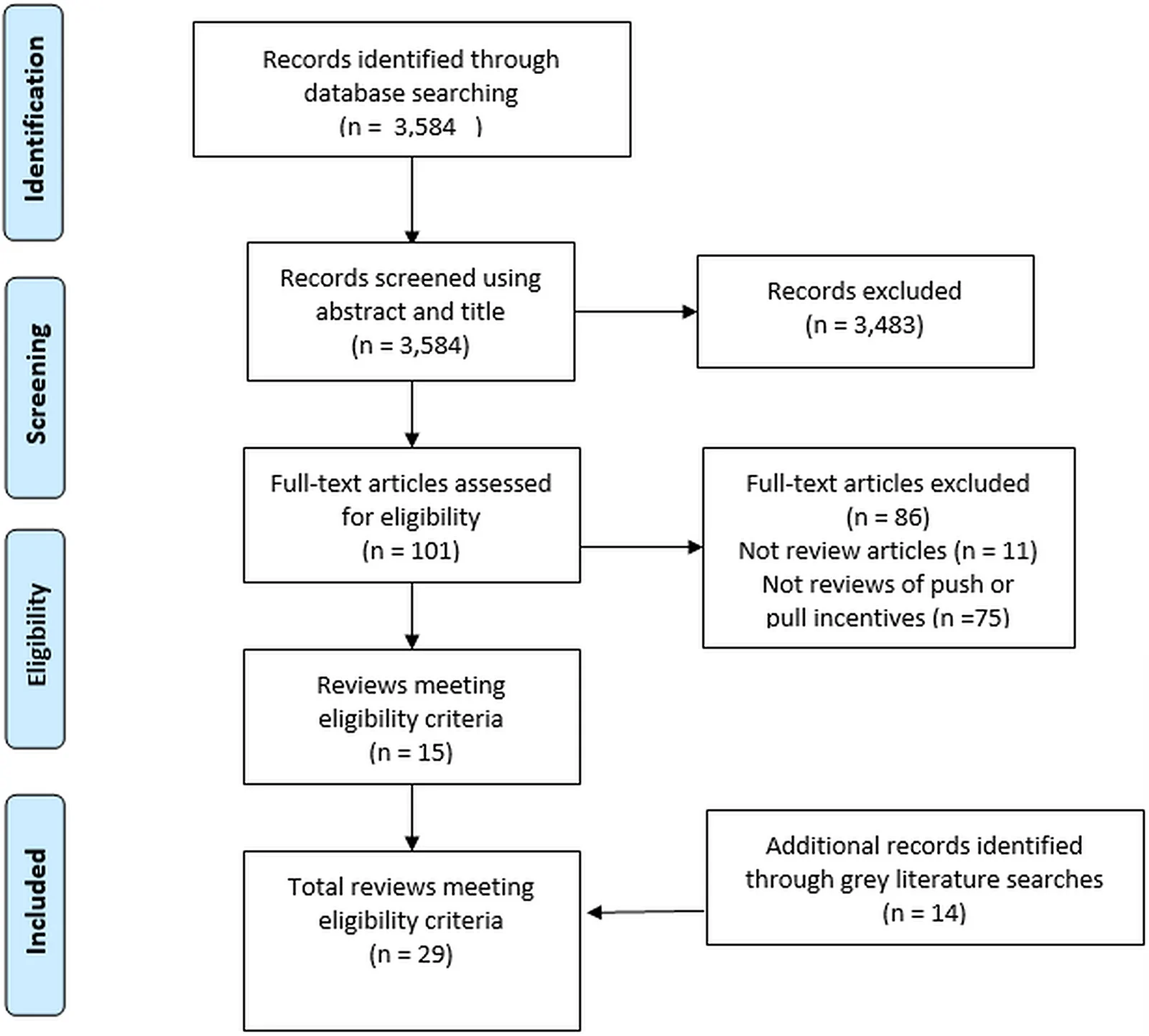
PRISMA diagram.

Our initial search for existing reviews of antibacterial innovation and access incentives yielded 3584 results (Fig. 1). After title and abstract screening, 101 reviews were identified for full-text screening. Following academic database screening, 15 reviews were identified from the full-text screening, and an additional 14 were identified through grey literature searches (Appendix Table S2). In total, 29 reviews of antibacterial innovation and access incentives published between 2014 and 2024 met our eligibility criteria.

**Fig. 2:**
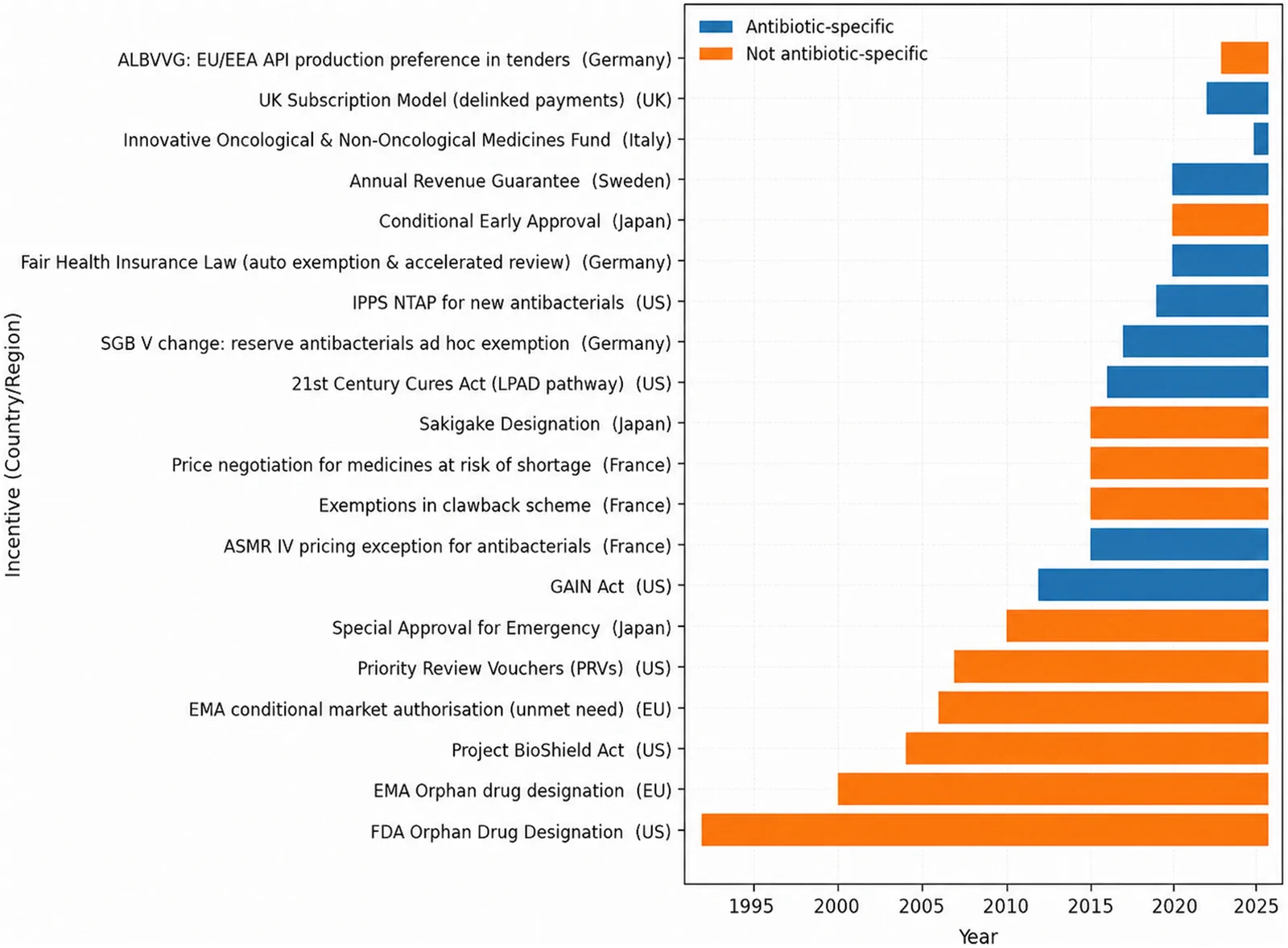
Timelines for implemented antibacterial pull incentive mechanisms. ALBVVG = Act to Combat Supply Shortages of Off-Patent Medicines and to Improve the Supply of Paediatric Medicines, IPPS NTAP = Inpatient Prospective Payment System—New Technology Add-on Payment, SGB V = Sozialgesetzbuch Fünftes Buch (German Social Code, Book V), LPAD = Limited Population Antibacterial Drug, GAIN = Generating Antibiotic Incentives Now, PRVs = Priority Review Vouchers, EMA = European Medicines Agency, FDA = U.S. Food and Drug Administration, ASMR = Amélioration du Service Médical Rendu.

We identified 28 pull incentives: 24 from existing reviews and 4 from grey literature searches (Appendix Table S3). Of these, 20 were implemented in the UK, Sweden, Germany, France, Italy, the US, Japan, and the EU; and 8 were proposed or in development in the US, Japan, Canada, Australia, Switzerland, and the EU. Fig. 2 shows time periods for incentives implemented. Pull incentives have evolved from broad regulatory and market measures to antibacterial-focused payment models. Early schemes, e.g. the Project BioShield (2004)^13^ and Priority Review Vouchers (2007),^14^ were not antibacterial-specific, targeting rare (i.e. rare paediatric diseases) or emergency products more broadly. These older, non-antibacterial-specific incentives and regulatory flexibilities have generally been regarded as insufficiently tailored to the antibiotic market and showed limited evidence of materially improving antibacterial R&D incentives or commercial sustainability.^15^ This helped motivate the development of antibacterial-specific pull mechanisms from the mid-2010s onward, such as France's pricing exceptions (2015),^16^ Germany's reference-pricing reforms (2017 onward),^17^ Sweden's annual revenue guarantee (2020),^18^ and the UK's subscription model (2022)—reflecting a shift toward tailored reimbursement and market-stabilising mechanisms for critical antibacterials.^19^

### Implemented pull incentives

Following our review of product and company eligibility and evaluation criteria, we summarised the common domains within implemented pull incentives (Table 2), and the pathogens targeted across implemented and in-development pull incentives (Table 3). The product and company eligibility and evaluation criteria for implemented pull incentives are contained within an open repository (https://github.com/mikeuk2024/antibioticincentives). As the regulatory reforms in France, Germany, US, and Japan were not mutually exclusive across the incentives identified, their eligibility and evaluation criteria were examined together. For instance, in Germany, the *Changes in §35 SGB V* and the *Fair Health Insurance Law* both link eligibility to designation as ‘reserve’ antibacterials.^17^ The 20 implemented pull incentives identified were therefore grouped into nine categories: the UK Subscription Model, the Swedish Annual Revenue Guarantee (ARG) Model, German reimbursement reforms, French reimbursement reforms, the Italian Innovative Medicines Fund, US regulatory reforms, US new technology add-on payments, Japanese regulatory reforms, and the EU conditional market authorisation framework.

**Table 2:** Product and company eligibility and evaluation criteria for implemented antibacterial pull incentives initiatives.^a^

	UK subscription model	Sweden ARG model	Germany reimbursement reforms^b^	France reimbursement reforms^c^	Italy innovative medicines fund	US regulatory reforms^d^	US New technology add on payments^e^	Japan regulatory reforms^f^	European union regulatory reforms^g^
Product eligibility and evaluation criteria									
Targeting high-priority medical need	Yes	Yes	Yes	Yes	Yes	Yes	Yes	Yes	Yes
Relative effectiveness	Yes	No	Yes	Yes	No	No	Yes	Yes	Yes
Unmet clinical need	Yes	Yes	Yes	Yes	No	Yes	No	Yes	Yes
Innovative characteristics	Yes	No	No	No	No	Yes	Yes	Yes	No
Health-system impact	Yes	Yes	No	Yes	No	No	Yes	No	No
Other product characteristics	Yes	No	No	Yes	No	No	Yes	Yes	No
Company eligibility and evaluation criteria									
Antibacterial sustainability	Yes	Yes	Yes	No	Yes	No	No	No	No
Patient access	Yes	Yes	Yes	No	Yes	No	No	No	No
Environmental health	Yes	Yes	No	No	No	No	No	No	No
Economic criteria	Yes	Yes	Yes	Yes	No	Yes	No	No	No
Other	Yes	Yes	No	No	No	Yes	No	Yes	Yes

a This table does not include pull incentives that are proposed, or in pilot phases. Annual Revenue Guarantee (ARG).

b Changes in 35 SGB V—exemption of ‘reserve’ antibacterials from internal reference groups. Fair Health Insurance Law—Automatic exemption from internal reference groups, and accelerated reimbursement review following EMA approval.

c Exemption from international reference pricing for antibacterials with ASMR level IV (minor) classification & exemptions from clawback scheme.

d Qualified Infectious Disease Product Designation (QIDP) for five years of additional market exclusivity, and faster regulatory review under the 2012 GAIN (Generating Antibiotic Incentives Now) Act. Limited Population Antibacterial Drug (LPAD) for faster regulatory review, and Priority Review Vouchers. FDA Orphan Drug Designation for antibacterials meeting rare disease and economic requirements. Project BioShield Act, establishing Emergency Use Authorisation for accelerated regulatory access to unapproved countermeasures during CBRN emergencies, alongside guaranteed federal procurement for qualifying countermeasures.

e Updates in Inpatient Prospective Payment System IPPS Rule–New technology add-on payments.

f Priority regulatory review, ‘premium pricing’, and extended data protection period for drugs meeting certain criteria.

g EMA Conditional Marketing Authorisation for unmet needs, and Orphan Drug Designation for antibacterials meeting rare disease and economic requirements.

**Table 3:** Target Pathogens across pull incentives.

WHO priority pathogen list 2024	RKI non-exhaustive list of multidrug-resistant bacteria (2024)^17^	Qualifying pathogens under the 2012 food and drug administration safety and innovation act^20^	Pathogens included in the 2024 antibacterial resistance threats in the United States' report^21^	Public Health Agency of Canada priority pathogens for the pilot project^22^
**Priority 1: Critical** • *Acinetobacter baumannii*, carbapenem-resistant • Enterobacterales, carbapenem-resistant • Enterobacterales, third generation cephalosporin-resistant • *Mycobacterium tuberculosis*, rifampicin-resistant **Priority 2: High** • *Enterococcus faecium*, vancomycin-resistant • *Neisseria gonorrhoeae*, third-generation cephalosporin, and/or fluoroquinolone-resistant • Non-typhoidal Salmonella, fluoroquinolone-resistant • *Pseudomonas aeruginosa*, carbapenem-resistant • Salmonella Typhi, fluoroquinolone-resistant • Shigella spp., fluoroquinolone-resistant • *Staphylococcus aureus*, methicillin-resistant **Priority 3: Medium** • Group A Streptococci, macrolide-resistant • Group B Streptococci, penicillin-resistant • *Haemophilus influenzae*, ampicillin-resistant • *Streptococcus pneumoniae*, macrolide-resistant	• *Acinetobacter baumannii*, CR • *Burkholderia cepacia* complex, CR • *Burkholderia cepacia* complex, 3GCR • Campylobacter spp., FQR • Citrobacter spp., 3GCR • Enterobacter spp., CR • Enterobacter spp., 3GCR • *Enterococcus faecium*, VR • *Escherichia coli*, CR • *Escherichia coli*, 3GCR • *Haemophilus influenzae*, AmpR • *Helicobacter pylori*, ClaR • Klebsiella spp., CR • Klebsiella spp., 3GCR • Morganella spp., 3GCR • *Mycobacterium tuberculosis*, RR • *Neisseria gonorrhoeae*, FQR • *Neisseria gonorrhoeae*, 3GCR • Non-typhoidal salmonella, FQR • Proteus spp., 3GCR • Providencia spp., 3GCR • *Pseudomonas aeruginosa*, CR • Serratia spp., 3GCR • Shigella spp, FQR • *Staphylococcus aureus*, MR • *Stenotrophomonas maltophilia*, FQ	• Acinetobacter species • Aspergillus species • *Burkholderia cepacia* complex • Campylobacter species • Candida species • *Clostridium difficile* • Coccidioides species • Cryptococcus species • Enterobacteriaceae (e.g. *Klebsiella pneumoniae*) • Enterococcus species • *Helicobacter pylori* • *Mycobacterium tuberculosis* complex • *Neisseria gonorrhoeae* • *N. meningitidis* • Non-tuberculous mycobacteria species • Pseudomonas species • *Staphylococcus aureus* • *Streptococcus agalactiae* • *S. pneumoniae* • *S. pyogenes* • *Vibrio cholerae*	• *Acinetobacter,* Carbapenem-resistant • *Candida auris* (*C. auris*) • Enterobacterales, Carbapenem-resistant (CRE) • Enterococcus, Vancomycin-resistant (VRE) • Extended-spectrum beta-lactamase (ESBL)-producing Enterobacterales • *Pseudomonas aeruginosa,* Multidrug-resistant (MDR) • *Staphylococcus aureus,* Methicillin-resistant (MRSA)	• *Acinetobacter baumannii* • Enterobacteriaceae • *Neisseria gonorrhoeae* • *Pseudomonas aeruginosa* • *Staphylococcus aureus*

AmpR = Ampicillin-resistant, CR = Carbapenem-resistant, ClaR = Clarithromycin-resistant, 3GCR = resistant against 3rd generation Cephalosporin, FQR = Fluoroquinolone-resistant, MR = Methicillin-resistant, RR = Rifampicin-resistant, VR = Vancomycin-resistant.

The mapping in Table 2 highlights both commonalities and divergences in how product and company criteria are applied. While targeting high-priority medical needs and unmet clinical need are widely included, there is marked variation in the inclusion of innovative characteristics, health-system impact, and environmental safeguards. Company-level obligations such as stewardship, patient access, and environmental health are more prominent in the UK and Sweden initiatives. Across the nine groups of implemented pull incentives reviewed, targeting high-priority medical need (100%, 9/9) and unmet clinical need (78%, 7/9) were the most consistently applied criteria. Relative effectiveness featured in 67% (6/9) and innovative characteristics in 44% (4/9), indicating moderate emphasis on comparative performance and novelty. Broader evaluative elements such as health-system impact and patient access appeared in 44% (4/9) of schemes, while economic criteria were referenced in 56% (5/9). In contrast, antibacterial sustainability and environmental health were explicitly incorporated in only 44% (4/9) and 22% (2/9) respectively. Overall, the UK Subscription Model (100%, 11/11) and Swedish Annual Revenue Guarantee (73%, 8/11) incorporated the widest range of criteria.

Table 3 shows that most pull incentives reference established priority pathogen lists, such as those from WHO, the US Food and Drug Administration (FDA), or national public health agencies, but the breadth and specificity of these lists differ significantly. Some countries focus narrowly on a small set of critical pathogens, while others adopt a broader list that includes medium-priority organisms or specific clinical syndromes. The country case studies that follow demonstrate how these pathogen lists are operationalised in different policy contexts, and how they interact with eligibility and evaluation criteria for different incentive options.

### UK subscription model

In the UK, the National Institute for Health and Care Excellence (NICE) and NHS England deploy a subscription payment model to encourage pharmaceutical companies to develop new antibacterials for resistant infections.^19^ Under the initial pilots, Pfizer and Shionogi submitted two novel cephalosporin antibacterials—ceftazidime/avibactam (Zavicefta®) and cefiderocol (Fetcroja®)—for assessment. In June 2022, the first two subscription contracts committed NHS England to pay up to £10 million annually for each antibacterial over an initial three-year period, with an option to extend up to ten years.^23^ The subscription model, incorporating the lessons learnt, has now been extended to include further antibacterials.

**Fig. 3:**
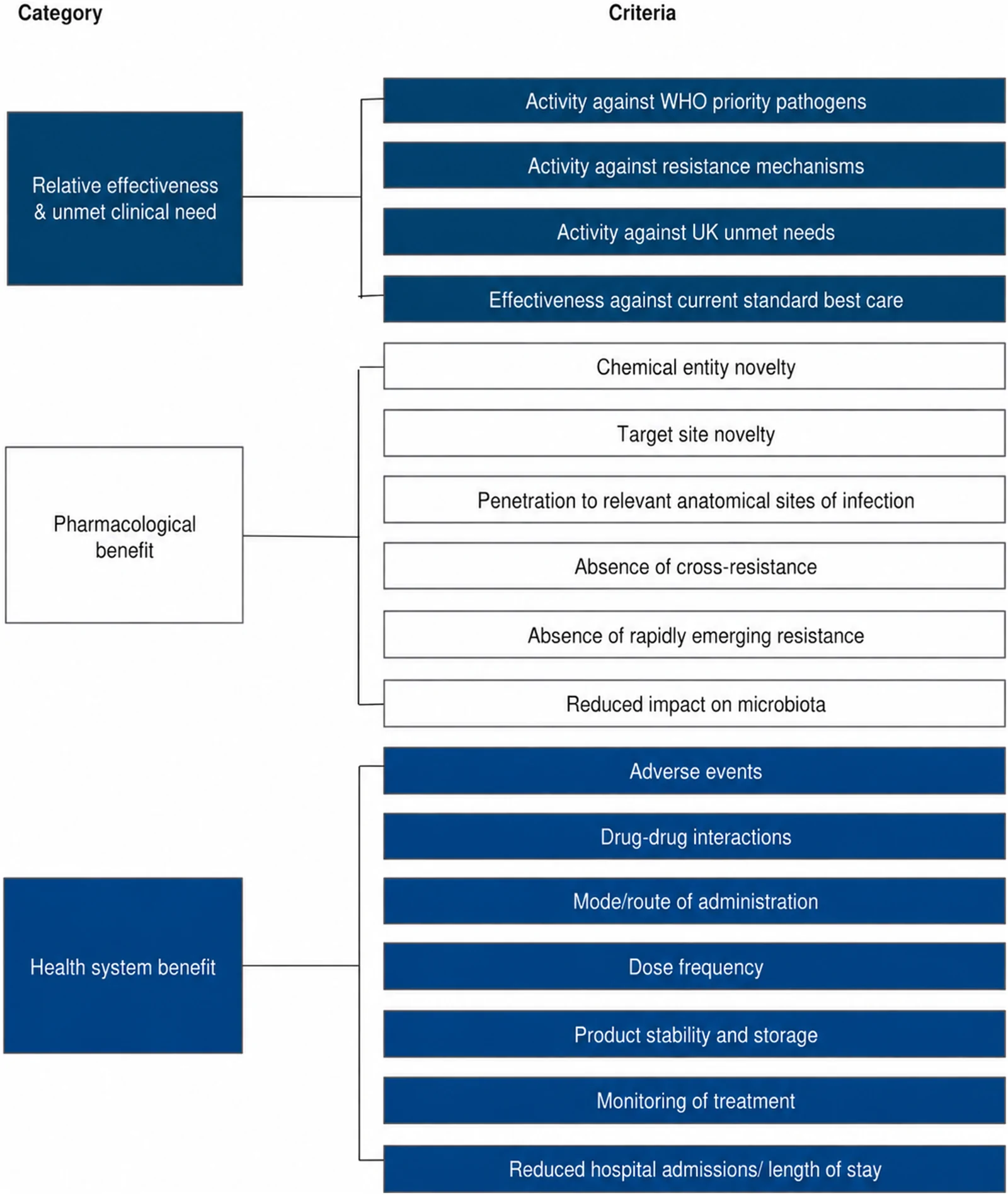
UK subscription payment award categories and criteria for antibacterial evaluation. Source: NHS England.^25^

To qualify for inclusion, products must receive authorisation from the Medicines and Healthcare products Regulatory Agency (MHRA), and be judged as meeting unmet clinical need, i.e. they target pathogens identified as critical by WHO.^24^ Once deemed eligible, the novel antibacterials are evaluated by NICE and allocated tiered annual payments between £5 million and £20 million from England, with additional contributions from Scotland, Wales, and Northern Ireland proportional to population size. NICE's award criteria fall into three areas: relative effectiveness and unmet clinical need, pharmacological benefit, and health system benefit; see Fig. 3 for sub-criteria.

To be deemed eligible to receive UK subscription payments, companies must: provide contractual assurances to maintain consistent supply of antibacterial products, and ensure availability to meet demand; promote responsible use of antibacterials, e.g. informative and educational promotion of products, delinking remuneration for antibacterial sales; facilitate independent assessment of compliance with specified antibacterial manufacturing standards; demonstrate their economic and financial standing is sufficient to justify award of the proposed contract; and demonstrate their commitment to specified social value requirements, e.g. achieving Net Zero carbon emissions.^26^

### Swedish annual revenue guarantee

In Sweden, the government piloted a revenue guarantee model to improve access to antibacterials of high medical value.^18^ By July 2020, the procurement phase concluded, with guaranteed annual payments of SEK 4 million (approximately €400,000) per product agreed for five antibacterials: ceftolozane/tazobactam (Zerbaxa®), imipenem/cilastatin/relebactam (Recarbrio®), cefiderocol (Fetcroja®), and meropenem/vaborbactam (Vaborem®); and one older antibacterial, fosfomycin in intravenous formulation, not previously marketed in Sweden.^27^

The criteria for inclusion in the pilot study were based on priorities set by the Public Health Agency of Sweden (PHAS) in collaboration with independent experts. PHAS's priorities aligned closely with the WHO critical priority pathogen list.^28^

The eligibility criteria also included: approval by the European Medicines Agency (EMA); proven efficacy against at least one of the following carbapenem-resistant pathogens: *Enterobacterales, Pseudomonas aeruginosa*, or *Acinetobacter baumannii*; regulatory approval for use where treatment options are limited (i.e. unmet clinical need) or for at least two of the following: complicated intra-abdominal infections, complicated urinary tract infections, hospital-acquired pneumonia; and a safety profile comparable to β-lactam antibacterials.

For companies, the Swedish model stipulates they must: be experienced in delivering services to the Swedish or European market: demonstrate the ability to deliver the volumes agreed in the contract (e.g. warehouse capacity, delivery capacity, and reporting mechanisms)^27^; have a warehouse located in Sweden; commit to requirements related to security of supply and availability, e.g. to dispatch orders for antibacterials to hospitals the next working day, to document and regularly report on warehouse stocks, sales volumes, distribution channels, and delivery times; and comply with environmental regulations to minimise the environmental impact of pharmaceutical production and disposal.^29^^,^^30^

### German reimbursement reforms

Reimbursement schemes in Germany to promote sustainable access to so-called ‘reserve’ antibacterials,^17^ include exemption for internal reference pricing, and early benefit assessment. ‘Reserve’ antibacterials can also have their initial sales price maintained beyond six months to encourage development and availability.^31^ Stewardship requirements exist for reserve antibiotics, with the Federal Joint Committee issuing quality assurance requirements for the pharmaceutical company's prescribing information shared with healthcare providers.^17^

Eligibility for classification as a ‘reserve’ antibacterial requires the antibacterial to target a pathogen on the German list of multi-drug-resistant bacteria (MDRB) (Table 3). This list was developed in four stages. First, ‘treatability’: pathogens classified by WHO as having limited or no treatment were included. Second, pathogens whose treatability is deemed sufficient by WHO were evaluated using additional criteria specific to Germany, e.g. transmissibility, preventability, mandatory notification, resistance proportion, and trend of resistance. Third, these scores were combined with expert opinion on clinical relevance to practice in Germany. Fourth, further pathogens, outside the WHO priority list, could be included based on expert opinion. The MDRB list is seen as a non-exhaustive list, and can change due to high clinical relevance and lack of treatment options.

Germany has also introduced measures to promote a sustainable supply of new and existing antibacterials between manufacturers and health insurers.^31^ Company eligibility criteria include priority for generic manufacturers which use active pharmaceutical ingredients in the EU or European Economic Area; reporting on stock levels, production sites, and sales volumes to prevent shortages; maintaining a three-month stockpile of generic medicines, including antibacterials.

### French reimbursement reforms

In France, reimbursement of new medicines is based upon added clinical value and overall clinical benefit as independently assessed by the Transparency Committee of the French National Authority for Health (TC-HAS).^32^ Added clinical value is determined by comparing existing and new products in terms of clinical efficacy, quality of life, and tolerance of the novel treatment and the methodological quality of the study (including choice of comparators, and the adequacy of the population included). Products are then assigned an ‘Amélioration du Service Médical Rendu (ASMR)’ rating (major—ASMR I, significant—ASMR II, moderate—ASMR III, minor—ASMR IV, or non-existent—ASMR V). Antibacterials with at least ‘minor’ added clinical value are eligible to preferential pricing, whereby market authorisation holders are guaranteed a price not lower than the lowest price across four reference countries.

Overall clinical benefit is ranked using four Service Médical Rendu (SMR) categories which have different reimbursement rates: high (65%), moderate (30%), low (15%) or insufficient (meaning that TC-HAS recommends not reimbursing the drug). Specific guidance on antimicrobial resistance from TC-HAS sets out five criteria for evaluating new antibacterials: the seriousness of the condition targeted; the antibacterial's efficacy and adverse effects; its place in the therapeutic strategy; its preventive, curative or symptomatic nature; its public health impact.^33^ Public health impact is examined in terms of improving the population health, addressing unmet clinical needs, or reducing resource consumption (i.e. health-system impact). TC-HAS emphasises that antibacterials targeting WHO priority pathogens will be assessed as improving population health and addressing unmet clinical needs.

New measures to improve supply of antibacterials include exemption of certain antibacterials from turnover clawback mechanisms. Companies may request permission for a price increase from the reimbursement authority if continued commercialisation would otherwise be unviable.^16^ To be eligible, companies must provide information regarding proposed selling price to healthcare providers, prices charged and forecast sales volumes to main states in EU, and prices charged to healthcare providers in France over the three previous years.

### Italian innovative medicines fund

In Italy, from 2025 onwards the Italian Medicines Agency (IMA) has been granted a dedicated budget of up to €100 million a year with which to negotiate potentially higher prices for new antibacterials.^34^ To be eligible, antibacterials must be classified as “Reserve” in the World Health Organization's (WHO) AWaRe list, or target a pathogen within the WHO Bacterial Priority Pathogens List (PPL).^35^ Unlike other medicines, reserve antibacterials are exempt from IMA's standard innovativeness assessment, though price and reimbursement are still negotiated through IMA's ordinary process.^35^ The antibacterial must be used for treating infections caused by multidrug-resistant bacteria, and be under patent or data protection. Eligible antibacterials will be exempt from Italy's pharmaceutical payback mechanisms within the constraints of the €100 million budget ceiling. Antibacterials within the scheme must be made immediately available to patients, even without formal inclusion in regional hospital therapeutic formularies. Usage will be monitored by the Italian Medicines Agency's registry system to support appropriate use and stewardship in Italy.^35^ As of January 2026, nine antibacterials have been classified as eligible for the scheme.^36^

### US regulatory reforms

In 2012, the FDA established the Qualified Infectious Disease Product (QIDP) designation under the Generating Antibiotic Incentives Now (GAIN) Act.^37^ The incentives include priority review and five additional years of market exclusivity for FDA-approved, QIDP-designated drugs. Eligibility for QIDP requires the product is an antibacterial or antifungal that has the capacity to treat serious or life-threatening infections caused by resistant pathogens, including novel or emerging pathogens, or qualifying pathogens outlined by the FDA (Table 3).^38^ A disease or condition is considered serious if it has a substantial impact on day-to-day functioning.^39^ This includes diseases that, if left untreated, progress to a more severe state or become life-threatening. A disease or condition is life-threatening if it has a high likelihood of death unless treated.^40^

To qualify for Limited Population Antibacterial Drug (LPAD) designation, an antibacterial or antifungal must meet the following criteria: the drug should aim to treat a serious or life-threatening infection (as defined above), and be intended for a limited population of patients with unmet clinical needs.^41^ The FDA defines an unmet clinical need as a situation in which no satisfactory therapy or treatment exists for a serious or life-threatening disease or condition. This can also apply to cases where current treatments are not adequate, i.e. they provide insufficient benefit, have unacceptable side effects, or are unsuitable for some patients with the condition.^42^

The FDA grants priority review vouchers (PRVs), which entitle the sponsor to a six-month review rather than the standard ten-month review, in several therapeutic areas relevant to antibacterials. PRVs were originally established for neglected tropical diseases from 2007 onwards, extended to rare paediatric diseases in 2012 and medical countermeasures to pathogens identified as a threat by the US Department of Homeland Security (DHS) in 2016. Eligibility for a PRV for neglected tropical diseases requires the therapy to prevent or treat a pathogen included in the FDA tropical disease list.^14^ Eligibility for a rare paediatric disease PRV requires the drug to target a disease that: (1) a serious or life threatening disease that primarily affects those aged 18 years or younger; (2) affects fewer than 200,000 people in the United States, or more but with no reasonable expectation of recovering development costs; and (3) has not previously been treated by an approved product containing the same active ingredient. Eligibility for a medical countermeasure PRV requires the therapy, or device to treat, prevent, or diagnose a condition caused by a chemical, biological, radiological, or nuclear (CBRN) threat, or a pathogen identified as a material threat by the US Department of Homeland Security (DHS). To date, PRVs have been granted to antibacterials targeting multidrug-resistant tuberculosis (MDR-TB). While some selected bacteria are included in scope as neglected tropical diseases,^14^ WHO priority pathogens are not included.

Antibacterials may also be eligible for FDA orphan drug designation if the antibacterial is considered as treating a rare disease (i.e. fewer than 200,000 people in the United States), and the sponsor is not expected to recover development and marketing costs plus reasonable profit within seven years of FDA approval.^43^ Orphan drug designation qualifies sponsors for incentives including tax credits for qualified clinical trials, exemption from user fees, potential seven years of market exclusivity after approval.

Mechanisms to improve security of supply and create a guaranteed market for antibacterials include the use of Project BioShield Act 2004 to put in place advance purchase agreements for antibacterials that are classified as responding to biological, chemical, radiological, or nuclear (CBRN) threats, e.g. anthrax.^13^ The Act also established Emergency Use Authorisation (EUA), allowing the FDA to permit use of an unapproved product, or an unapproved use of an approved product, during a declared public health emergency. This has been used to put in place a contract between BARDA and Paratek Pharmaceuticals to secure stockpiles of Omadacycline that can be used against anthrax, invoking both the Act's procurement guarantee and its EUA provision.^44^

### US new technology add-on payments (NTAPs)

New technology add-on payments (NTAPs) granted by the Centers for Medicare and Medicaid Services (CMS) are not specifically for antibacterials but can be used for them if they meet three criteria. The antibacterials must: (1) represent a substantial clinical improvement; (2) be new and not substantially similar to an existing technology; and (3) be relatively costly such that the diagnosis-related group (DRG) payment rate otherwise applicable would be inadequate. Starting in the fiscal year (FY) 2021 application cycle, CMS will consider QIDPs as new and not substantially similar to an existing technology, and they will not need to meet the requirement to represent a substantial clinical improvement.^45^ This is an antibacterial-specific amendment to the NTAPs.

### Japan regulatory reforms

Three main regulatory reforms have facilitated accelerated regulatory review, market entry, and premium pricing for novel medicines in Japan. The Sakigake Designation System, introduced in 2015, requires products to meet three criteria: (1) they must introduce a novel mechanism compared to existing therapeutic options; (2) preliminary clinical or non-clinical data must suggest that the product offers a step-change in terms of effectiveness; and (3) must target a serious or life-threatening condition with high unmet clinical needs and limited or no treatment options. Company eligibility criteria include a commitment to seek approval for the product in Japan ahead of or simultaneously with other countries.^46^

The Conditional Early Approval System, introduced in 2017, enables accelerated regulatory review and early market entry for drugs with preliminary evidence of clinical benefits that require further evidence of safety and efficacy.^46^ First, as in the Sakigake Designation System, the product must target life-threatening diseases or irreversible progressive diseases that significantly impair daily living activities. Second, there must be no other treatment options or the product is expected to substantially improve patient outcomes. Third, there must be challenges in conducting further clinical trials, e.g. a limited number of patients and small populations. Companies must also comply with post-marketing surveillance of efficacy and safety.

The Special Approval for Emergency System, introduced in 2010, permits rapid regulatory review and approval, with additional post-marketing data requirements, for drugs intended to prevent the spread of disease during a public health emergency.^46^ Eligibility requires that no alternative treatment is available in Japan and that the product has already been approved in a foreign country with a robust regulatory system; unlike Sakigake and the Conditional Early Approval System, novelty and comparative effectiveness are not assessed.

### European union

The European Medicines Agency (EMA) offers conditional market authorisation as a pathway to accelerate access to novel antibacterials that address unmet clinical needs, e.g. infections caused by multidrug-resistant pathogens.^47^ To be eligible, the antibacterial's manufacturer must show that anticipated benefits outweigh potential risks, even if the clinical data is incomplete at the time of approval; and provide additional clinical data post-authorisation to confirm efficacy and safety. Faster regulatory approval is justified when the antibacterial is likely to meaningfully improve patient outcomes or benefit public health.

The EMA also offers orphan drug designation to eligible antibacterials, which allows up to ten years of market exclusivity following approval, reduced regulatory fees, and tailored scientific support through the regulatory approval process.^48^ To be eligible, the antibacterial must: (1) address a life-threatening or chronically debilitating condition; (2) affect no more than 5 in 10,000 people in the EU, or demonstrate insufficient return on investment is likely even if prevalence is higher; and (3) provide a significant benefit over existing options, or there must be limited existing treatment options available.

### Proposed or in-development pull incentives

We identified several pull incentives proposed or under development in Japan, the US, Canada, the EU, Australia, and Switzerland. These included mechanisms such as revenue guarantees, subscription-based payments, transferable exclusivity extensions, and modified health technology assessment (HTA) or reimbursement pathways. In Supplementary Material (page 13 onwards), we describe the provisional information available on eligibility and evaluation criteria for these proposed incentives, although such information remains subject to change as initiatives progress.

## Discussion

This review identified 28 implemented or proposed pull incentive initiatives aimed at stimulating investment in R&D, launch, equitable access, and sustainable antibacterial markets. Many have emerged in the past six years, reflecting growing policy interest in sustainable antibacterial innovation and access. The most frequently adopted product-related criteria for inclusion in a pull incentive are: targeting high-priority medical need, unmet clinical need, and relative effectiveness. Company-related criteria were less consistently applied and, when present, focused on stewardship, equitable access, supply security, and environmental safeguards.

UK and Sweden stand out for applying multiple company-level obligations in addition to product eligibility and evaluation criteria to their pull incentives. In contrast, incentives in France, and the US are product-focused, with fewer company obligations. The difference reflects the nature of the pull incentives in UK and Sweden: both utilise annual revenue guarantees that provide leverage to impose company-level conditions such as supply security, good stewardship, and environmental health protection. Identified pull incentives demonstrated variation in pathogen prioritisation, with some countries adopting target lists aligned closely to WHO's critical pathogens, and others using broader or nationally specific lists. This may reflect differences in public health priorities and epidemiological trends across countries.

Overall, there was considerable variation across national models in both the presence and specificity of product and company eligibility and evaluation criteria. Narrow and conflicting criteria risks giving mixed signals to developers and may undermine global promotion of antibacterial innovation and access. This also contributes to uncertainty around returns on antibacterial R&D, which may discourage funders from allocating investments compared with other therapeutic areas. These findings highlight the need to build consensus and identify a core set of shared priorities and criteria globally—allowing for flexibility to reflect different country contexts, public health needs, and incentive objectives—in order to maximise the impact and efficiency of antibacterial pull incentives.

The major strength of this review is its comprehensive and timely analysis of product and company eligibility and evaluation criteria for global pull incentives, integrating both comparative synthesis (Tables 2 and 3) and country case studies. These findings can lay the groundwork for future consensus building, using expert panels to refine and prioritise criteria globally. By deploying an umbrella review approach and drawing from academic and grey literature to identify relevant pull incentives, we have applied a systematic protocol to minimise the risk of overlooking relevant policies and mechanisms.

Nonetheless, our analysis has limitations. As with any review relying on publicly available data, some eligibility and evaluation criteria, particularly those embedded in confidential contracts, remain inaccessible. While incorporating double verification into our data collection strengthens our methodology, there are still risks that information was overlooked, particularly in countries without English language documentation. We were unable to comment on the relative effectiveness of different mechanisms. In many cases, it remains too early to evaluate their comparative effectiveness in promoting R&D for novel antibacterials due to the length of time required to bring antibacterial candidates through the basic science, preclinical, and clinical research stages. It also was not possible to consistently collect information on the size of incentive as this information was often not publicly available or subject to confidentiality agreements. Fourth, we were unable to differentiate between pull incentives according to whether their objectives comprised antibacterial R&D, or access to new and existing antibacterials. This is because in nearly all cases the stated objectives of identified pull incentives were both stimulating innovation and access, even when the size of incentive was relatively small and therefore only likely to have a meaningful impact on access. Finally, our analysis of stewardship, access, and environmental health criteria among pull incentives did not distinguish between those requiring only standards be met in sponsoring countries or globally, which is particularly important given the transnational nature of antibacterial supply-chains and usage.

The report's findings carry several important policy implications for governments, funders, and international organisations involved in antibacterial innovation and access. The heterogeneity in scope and detail of product and company eligibility and evaluation criteria across implemented pull incentives underscores the need for greater international coordination. In certain cases, variation may be warranted and reflect countries' local needs and priorities, in particular for schemes that aim to strengthen access to essential antibacterials in short supply in specific markets. In other cases, such as pull incentives that target R&D of new antibacterials, variation may be less justified. Policymakers could prioritise harmonising eligibility frameworks for antibacterial innovation and access incentives where appropriate, building on best practice, to develop a shared set of principles that can guide incentive design globally. Platforms such as the AMR R&D Hub,^9^ as well as multilateral fora including the G7 and G20, could play an important role in facilitating dialogue, information sharing, and coordinated approaches to incentive mechanism design. Some existing criteria, such as the stewardship and access requirements developed by the Combating Antibiotic-Resistant Bacteria Accelerator (CARB-X) could inform discussions and provide a starting point for developing a global stewardship and access standard.^49^ Increasingly, there is a move away from focussing primarily on priority pathogens to target product profiles (TPPs) that define desired pharmacological, clinical, and public health outcomes, such as activity against novel mechanism of action, in vitro activity, pharmacokinetics, route of administration, product stability and storage, and efficacy. Recently, the WHO has published TPPs for new antibacterial agents that will influence eligibility and evaluation criteria for pull incentives going forward.^50^

Alongside eligibility and evaluation criteria, these multilateral fora should encourage agreement on the appropriate size of pull incentives. Modelling has been done in this regard for an effective set of pull incentives for antibacterial R&D, which indicates the G7+EU27 low-end, mid-range, and high-end annual revenue targets are US$258, US$363, and US$562 million in global revenues (USD 2024).^51^ The same analysis emphasised the UK and Italy were the only G7+EU27 countries to meet these mid-range annual targets: the UK due to its subscription model, and Italy due to its higher use and now also its Innovative Medicines Fund.

In developing a shared set of priorities for incentive eligibility and evaluation criteria, it is important to acknowledge the inherent tension between wide-ranging criteria and operational feasibility. While the UK and Swedish models demonstrate that extensive criteria can address multiple policy objectives simultaneously, such comprehensiveness increases administrative complexity for both regulators and companies, potentially creating barriers, particularly for smaller developers with limited capacity. In contrast to the UK and Swedish approach, the Italian model only has a limited set of criteria primarily focused on priority pathogens and “Reserve” antibiotic status. As a result, implementation of the Italian model has been relatively straightforward and achieved within a few months. A compromise between these two approaches may be to distinguish between essential criteria that should apply universally (such as targeting priority pathogens and basic stewardship requirements) and supplementary criteria that can be implemented by countries with greater regulatory capacity, or applied progressively as monitoring systems mature.

In terms of future research priorities, there is potential to build upon the work already done, using consensus-building methods such as Delphi panels to prioritise criteria based on expert input and stakeholder perspectives. Such efforts should also assess the feasibility of implementing and enforcing different criteria across health systems, including stewardship, infection prevention, and control, and environmental health requirements, as well as the data infrastructure needed to monitor compliance and support effective governance. Future work on this topic could broaden its scope to include antifungals, antivirals, and other antimicrobial modalities (e.g. bacteriophages and antibody-based therapies).^52^ Finally, a mapping exercise of push incentives could be undertaken to complement this analysis to identify to what extent alignment exists between eligibility and evaluation criteria for push and pull incentives. This matters because both mechanisms should send clear, and consistent signals to developers.

### Conclusion

This review provides the most up-to-date global comparative analysis of pull incentive schemes for antibacterial innovation and access. The marked variation in product and company eligibility and evaluation criteria risks sending inconsistent signals to developers, fragmenting markets, and weakening the overall impact of these mechanisms. Greater alignment of the criteria, alongside clearer linkage between pathogen priorities and incentive terms, could improve efficiency, strengthen stewardship and access provisions, and ensure that public investment drives the development of antibacterials with the greatest public health value.

## Contributors

MA and EM drafted the manuscript, and contributed to study design and conception. MA and AC undertook the literature review on incentives. MA and ZC undertook data collection. All other authors provided comments and edits to iterative versions of the manuscript.

## Data sharing statement

This analysis relied upon publicly available information which is referenced throughout and freely available.

## Declaration of interests

WC is employed by Shionogi UK as Market Access & Government Affairs Director. NC is employed by the National Institute for Health and Care Excellence as Chief Scientific Officer. DP is employed by the AMR Action Fund as Director of International Policy. MA declares receiving fees from Simon Kucher for delivering a presentation on antibiotic incentives. Remaining authors declare no relevant conflicts of interest.
