# Supplementary material for: Designing eligibility and evaluation criteria for antibacterial pull incentives: a comparative review of implemented and emerging initiatives
Source: Lancet Reg Health Eur. 2026 Sep 4;70:101849. doi: 10.1016/j.lanepe.2026.101849 (PMC13572082; doi:10.1016/j.lanepe.2026.101849)
Supplement: Supplementary file 1 [file mmc1.docx]

Contents

[Appendix Table 1: Search Strategy for Medline and EMBASE	2](#_Toc230247559)

[Appendix Table 2: Reviews of Antibacterial Innovation and Access Incentives	3](#_Toc230247560)

[Appendix Table 3: Summary of Antibacterial Pull Incentives from existing reviews	9](#_Toc230247561)

[Product and Company Eligibility and Evaluation Criteria for Proposed or Under Development Pull Incentives	13](#_Toc230247562)

[References	17](#_Toc230247563)

# Appendix Table 1: Search Strategy for Medline and EMBASE

| Number | Search Terms | Results |
| --- | --- | --- |
| 1 | ("antibacterial*" or "antibacterial*" or "antibacterial*" or "antifungal*" or "antiinfective*" or "anti-biotic" or "anti-microbial" or "anti-bacterial" or "anti-fungal" or "anti-bacterial" or "anti-infective" or "anti-biotics" or "anti-microbials" or "anti-bacterials" or "anti-fungals" or "anti-infectives").tw. | 1554787 |
| 2 | ("incentive*" or "policy" or "policies" or "stimulus" or "reimbursement" or "payment" or "regulation" or "fund" or "contract*" or "invest*").tw. | 13073118 |
| 3 | ("research" or "development" or "access" or "clinical trial" or "clinical trials" or "clinical study" or "clinical studies" or "preclinical trial" or "preclinical trials" or "preclinical study" or "preclinical studies" or "preclinical trial" or "preclinical trials" or "preclinical study" or "preclinical studies").tw. | 11810494 |
| 4 | 1 AND 2 AND 3 | 67045 |
| 5 | Limit 4 to (english language and yr="2014 - 2024") | 46887 |
| 6 | exp animals/ not humans.sh. | 36813585 |
| 7 | 5 not 6 | 22189 |
| 8 | Conference abstract.pt | 5250677 |
| 9 | 7 not 8 | 21678 |
| 10 | De-duplicate | 17481 |
| 11 | Filter by review | 3584 |

# Appendix Table 2: Reviews of Antibacterial Innovation and Access Incentives

| Review | Title | Focus | Pull incentives Identified |
| --- | --- | --- | --- |
| 1. So and Shah 2014 ^1^ | New business models for antibacterial innovation | Global |  |
| 2. Payne et al 2015^2^ | Time for a change: addressing R&D and commercialization challenges for antibacterials | Global |  |
| 3. Ardal et al. 2016^3^ | International cooperation to improve access to and sustain effectiveness of antibacterials | Global |  |
| 4. Kostyanev et al 2016^4^ | The Innovative Medicines Initiative’s New Drugs for Bad Bugs  programme: European public–private partnerships for the development of new strategies to tackle antibacterial resistance | EU |  |
| 5. Laxminarayan et al 2024^5^ | Expanding antibacterial, vaccine, and diagnostics development  and access to tackle antibacterial resistance | Global | - 2012 Generating Antibacterial Incentives Now (GAIN) Act - 21st century cures act 2016 - UK subscription model - PASTEUR Act |
| 6. McClelland et al 2021^6^ | Trends in antibacterial resistance legislation 2011-2019: A review of the US policy response to the antibacterial resistance threat and its public health Impact. | US | - Food and Drug Administration Safety and Innovation Act 2012 (this included GAIN) - 21st Century Cures Act 2016 - Pandemic and All-Hazards Preparedness and Advancing Innovations Act (PAHPAIA) of 2019 |
| 7. Ohashi et al 2022^7^ | A narrative review on drug development for the management of antibacterial- resistant infection crisis in Japan: the past, present, and future | Japan | - Sakigake Designation System - Conditional Early Approval System - Special Approval for Emergency System |
| 8. Outterson and Rex 2020^8^ | Evaluating for-profit public benefit corporations as an additional structure for antibacterial development and commercialization | Global | - UK subscription model - Swedish subscription model - Medicare FY2020 IPPS Final Rule |
| 9. Piddock et al 2024^9^ | Advancing global antibacterial research, development and access | Global | - Generating Antibacterial Incentives Now (GAIN) Act, - UK subscription model - Pioneering - Antibacterial Subscriptions to End Upsurging Resistance - (PASTEUR) Act) - a Japanese pilot subscription model - incentive program (which does not yet incentivize R&D), - Canadian pilot model (to be developed) - Transferable voucher model (EU) - WHO-SECURE initiative |
| 10. Renwick et al 2016^10^ | A systematic review and critical assessment of incentive strategies for discovery and development  of novel antibacterials | Global | - The GAIN Act - Limited Population Antibacterial Drug approval (US) - Priority review vouchers (us) - New technology add-on payment (us) - DISARM Act - 21st Century Cures Act - Project BioShield Act |
| 11. Sciarretta et al 2016^11^ | Economic Incentives for Antibacterial Drug Development: Literature Review and Considerations From the Transatlantic Task Force on Antibacterial Resistance | Global |  |
| 12. Simpkin et al 2017^12^ | Incentivising innovation in antibacterial drug discovery and development: progress, challenges and next steps | Global | - EMA - conditional market authorisation for drugs meeting unmet clinical need - 2012 GAIN ACT - 21st century cure act (limited population antibacterial drug programme) |
| 13. Singer et al 2020^13^ | (Inter)nationalising the antibacterial research and development  pipeline | Global | - UK subscription model |
| 14. Sinha and Kesselheim 2016^14^ | Regulatory Incentives for Antibacterial Drug Development: A Review of Recent Proposals | Global | - 21st Century Cures Act (limited population pathway) - 2018 DISARM Act (new technology add on payment) - The GAIN Act of 2012 (Qualified Infectious Disease Products (QIDPs)) - Priority Review Vouchers (PRVs) |
| 15. Vogler et al 2024^15^ | Pricing, Procurement and Reimbursement Policies for Incentivizing Market Entry of Novel Antibacterials and Diagnostics: Learnings from 10 Countries Globally | Australia, Brazil, France, Germany, Italy, Saudi Arabia, South Africa, South Korea, Spain, and Türkiye | - Spain- Innovation (including of novel antibacterials) may be considered in the pricing decision - Australia - Procurement of non-formulary medicines, including high-cost medicines and those for reserved treatment, e.g., antibacterials, based on a defined approval process - Germany -lower evidence requirements for reimbursement in - Exemption from HTA for defined reserve antibacterials. Tendering of health insurers for generic medicines with ‘open-house contracts’ for all interested suppliers. Antibacterials with API production in the EU/EEA to be considered in tendering of health insurers (2023 legislation) |
| 16. Anderson et al 2023^16^ | How can the EU support sustainable innovation and access to effective antibacterials? | EU | - UK subscription model - Sweden subscription model - Transferable exclusivity extension (not implemented) - Options model for antibacterials (not implemented) - Milestone payments (not implemented) - Market Entry Reward (not implemented) - GAIN Act – Market Exclusivity Extension - Gotham et al referenced (see below) |
| 17. Anderson et al 2023^17^ | Challenges and opportunities for incentivising antibacterial research and development in Europe | EU | - UK subscription model - Sweden subscription model - France- Exception for antibacterials with ASMR level IV (minor), and those assessed by non-inferiority clinical trials - Germany - Ad hoc exception of antibacterials from internal price reference groups and Automatic exception of ‘reserve’ antibacterials from internal price reference groups, accelerated reimbursement review process following EMA approval |
| 18. Gotham et al 2021 ^18^ | Reimbursement models to tackle market failures for antibacterials: Approaches taken in France, Germany, Sweden, the United Kingdom, and the United States | France, Germany, Sweden, UK, and the US | - Exception for antibacterials with ASMR level IV (minor) - France - Exemptions in clawback scheme (France) - Price negotiation for medicines at risk of shortage (France) - Changes in 35 SGB VAdhoc exemption of reserve antibacterials from internal reference groups (germany) - Fair Health Insurance Law - Automatic exemption from internal reference groups, and accelerated reimbursement review following EMA approval (Germany) - Sweden subscription model - UK subscription model - 2012 GAIN ACT - Updates in CMS Inpatient Prospective Payment System IPPS Rule (Increased reimbursement of cost to hospitals for procurement of newer antibacterials) - 2018 DISARM Act - PASTEUR |
| 19. Renwick et al 2016^19^ | Targeting innovation in antibacterial drug discovery and development: The need for a One Health – One Europe – One World Framework | Global | - GAIN Act |
| 20. Renwick et al 2014^20^ | A Critical Assessment of Incentive Strategies for Development of  Novel Antibacterials | Global | - GAIN Act - Limited Population Antibacterial (LPAD) approval - Priority review vouchers - New technology add-on payment (NTAP) - DISARM Act (not passed) - Project BioShield Act |
| 21. Kelly et al 2016^21^ | Public funding for research on antibacterial resistance in the  JPIAMR countries, the European Commission, and related European Union agencies: a systematic observational analysis | EU |  |
| 22. Ardal et al 2018^22^ | Revitalizing the antibacterial pipeline: Stimulating innovation while driving sustainable use and global access | Global | - GAIN |
| 23. Ardal et al 2017^23^ | Pull Incentives for Antibacterial Drug Development: An Analysis by the Transatlantic Task Force on Antibacterial  Resistance | Global | - GAIN Act - MER (not implemented) - TEE (not implemented) - Options model for antibacterials (not implemented) |
| 24. Dutescu and Hillier 2021^24^ | Encouraging the Development of New Antibacterials: Are Financial Incentives the Right Way Forward? A Systematic Review and Case Study | Global | - GAIN Act - UK Subscription model - Sweden subscription model - REVAMP Act (not implemented) |
| 25. Bhavnani et al 2020^25^ | A Broken Antibacterial Market: Review of Strategies to Incentivize Drug Development | Global | - GAIN Act - Medicare NTAP |
| 26. Savic and Ardal 2020^26^ | A Grant Framework as a Push Incentive to Stimulate Research and Development of New Antibacterials | Global |  |
| 27. Wasan et al 2023 | Landscape of Push Funding in Antibacterial Research: Current  Status and Way Forward^27^ | Global |  |
| 28. Cama et al 2021^28^ | To Push or To Pull? In a Post-COVID World, Supporting and Incentivizing Antibacterial Drug Development Must Become a Governmental Priority | Global | - Updates in CMS Inpatient Prospective Payment System IPPS Rule (Increased reimbursement of cost to hospitals for procurement of newer antibacterials) - UK Subscription model - Options Model for Antibacterials (proposed) |
| 29. Mattar et al 2020^29^ | An overview of the global antibacterial resistance research and development hub and the current landscape | Global |  |

# Appendix Table 3: Summary of Antibacterial Pull Incentives from existing reviews

| Pull Incentive | Country or Region | Mechanism Type | Years operational | Antibacterial Specific | Literature source |
| --- | --- | --- | --- | --- | --- |
| 1. Health Technology Assessment (HTA) Policy and Methods Review Reference Committee | Australia | Proposed pilot subscription fund for novel antibacterials | Proposed | Yes | Grey literature |
| 2. Canada's Antibacterial Economic Incentives Pilot Project | Canada | Intended to be a subscription model for antibacterials of interest and priority pathogens for the pilot project | Proposed | Yes | Grey literature |
| 3. EMA - conditional market authorisation for drugs meeting unmet clinical need | EU | Faster regulatory approval subject to additional data requirements for medicines that meet unmet clinical need (not specific to antibacterials) | 2006-present | No | Academic literature |
| 4. EU-level annual revenue guarantee model:    a. HERA- pilot annual revenue guarantee model    b. Revisions to EU pharmaceutical legislation include voluntary subscription model | EU | Annual revenue guarantee model for priority antibacterials at risk of inconsistent access across EU | Proposed | Yes | Academic literature |
| 5. Transferable exclusivity extension (TEE) | EU | Market authorisation holder of antibacterial successfully brough to market is granted a transferable voucher which can be used to extend market exclusivity for up to 12 months for any product in any therapeutic area | Revision agreed – but not yet granted to an antibacterial | Yes | Academic literature |
| 6. Exception for antibacterials with ASMR level IV (minor) - | France | Medicines with’ moderate’ or higher added therapeutic benefit are guaranteed a price not lower than the lowest price across 4 reference countries. This is extended to antibacterials with ‘minor’ added therapeutic benefit. | 2015- present | Yes | Academic literature |
| 7. Exemptions in clawback scheme | France | Sales of certain medicines exempted from turnover liable to clawback | 2015- present | No | Academic literature |
| 8. Price negotiation for medicines at risk of shortage | France | Companies may request permission for a price increase from the reimbursement authority, if continued commercialisation would otherwise not be viable | 2015- present | No | Academic literature |
| 9. Changes in 35 SGB V | Germany | Adhoc exemption of "reserve" antibacterials from internal reference groups | 2017- present | Yes | Academic literature |
| 10. Fair Health Insurance Law - | Germany | Automatic exemption from internal reference groups, and accelerated reimbursement review following EMA approval | 2020- present | Yes | Academic literature |
| 11. Act to Combat Supply Shortages of Off-Patent Medicines and to Improve the Supply of Paediatric Medicines (ALBVVG) | Germany | Antibacterials with API production in the EU/EEA to be considered in tendering of health insurers (2023 legislation) | 2023-present | No | Academic literature |
| 12. Innovative Oncological and Non-Oncological Medicines Fund | Italy | €EUR 100 million a year will be available to cover the reimbursement of these important antibacterials | 2025-present | No | Grey  literature |
| 13. Pilot Subscription Model | Japan | Pilot project for an annual revenue guarantee system for selection antibacterials | 2023 (Pilot only) | Yes | Academic literature |
| 14. Sakigake Designation System | Japan | Priority regulatory review, “premium pricing” and extended data protection period for drugs meeting certain criteria | 2015-present | No | Academic literature |
| 15. Conditional Early Approval System | Japan | Accelerated regulatory review and approval with additional data requirements | 2020-present | No | Academic literature |
| 16. Special Approval for Emergency System | Japan | Rapid regulatory review and approval with additional data requirements for drugs to prevent spread of disease, with no other drug available, and approved in a foreign country with a robust regulatory system. | 2010-present | No | Academic literature |
| 17. Annual Revenue Guarantee | Sweden | PHAS sets a minimum guaranteed annual revenue for selected originator antibacterials, in exchange for a guaranteed supply volume | 2020-present | Yes | Academic literature |
| 18. Swiss Roundtable on Antibacterials | Switzerland | Proposed revisions to the Swiss Epidemics Act | Proposed | Yes | Grey  literature |
| 19. UK Subscription Model | UK | Annual fee, negotiated based on AMR-specific HTA, delinked from volume supplied | 2022-present | Yes | Academic literature |
| 20. 2012 Generating Antibacterial Incentives Now (GAIN) Act | US | 5 years of additional market exclusivity, faster regulatory review | 2012-present | Yes | Academic literature |
| 21. 21st century cures act 2016 | US | Included Limited Population Antibacterial Drug (LPAD) approval. a regulatory pathway for the accelerated approval of new antibacterial and antifungal drugs | 2016-present | Yes | Academic literature |
| 22. Pioneering Antibacterial Subscriptions to End Upsurging Resistance (PASTEUR) Act | US | Contracts granted to selected new antibacterials guaranteeing minimum guaranteed revenues over 5+ years | Proposed, still under discussion | Yes | Academic literature |
| 23. Priority Review Vouchers (PRVs) | US | Vouchers that can be used to speed up the FDA review process for drugs that treat rare paediatric diseases, tropical diseases, or medical countermeasures (not specific for antibacterials) | 2007-2024 | No | Academic literature |
| 24. Updates in Inpatient Prospective Payment System IPPS Rule - New technology add-on payments | US | Increased reimbursement of cost to hospitals for procurement of newer antibacterials | 2019-present | Yes | Academic literature |
| 25. Developing an Innovative Strategy for Antibacterial Resistant Microbes (DISARM) Act | US | Reimbursement of antibacterial expenditures separately from general in-patient expenditures | Proposed, still under discussion | Yes | Academic literature |
| 26. Project BioShield Act | US | Provides a guaranteed market for companies that develop antibacterials and other qualified countermeasures. | 2004-present | No | Academic literature |
| 27. FDA- Orphan drug designation | US | Faster regulatory approval subject to additional data requirements for medicines that meet unmet clinical need (not specific to antibacterials) | 1992-present | No | Academic literature |
| 28. EMA Orphan drug designation | EU | Provides reduced regulatory fees, tailored scientific advice, and 10 years of market exclusivity following marketing authorisation. | 2000 - present | No | Academic literature |

# Product and Company Eligibility and Evaluation Criteria for Proposed or Under Development Pull Incentives

2.1.1. *Japan*

The Ministry of Health, Labour & Welfare (MHLW) in Japan has published plans to pilot an annual revenue guarantee model for antibacterials,^30^ but it is unclear which. The MHLW has produced a priority pathogen list by reviewing WHO and Centers for Disease Control and Prevention (CDC) lists, and categorising pathogens from priority 1 to 3, considering case fatality rate, community burden, resistance rates, trends in resistance rates, treatment options, and host response. Products are only eligible if there are limited or no treatment options available for the priority pathogen targeted. ^30^

For companies to be eligible, they must regularly collect data on sales volumes and report this information at the end of each financial year, and commit to appropriate use policies.^30^ The MHLW and Japan Agency for Medical Research and Development (AMED) intend to monitor appropriate use by monitoring prescriptions and ensuring guidance is followed. Companies must also commit to investing revenues generated above and beyond the annual revenue guarantee in R&D for new antibacterials.

2.1.2. *United States*

There are two proposed pull incentives in the US that have not been implemented. The Developing an Innovative Strategy for Antibacterial Resistant Microorganisms (DISARM) Act, first introduced in 2015, is a US legislative proposal aimed at enhancing Medicare reimbursement for certain antibacterial drugs. The act has never been passed, but intended eligibility criteria included new antibacterials approved by the FDA and designated as a QIDP (see section 3.2.5.).^31^

The Pioneering Antibacterial Subscriptions to End Upsurging Resistance (PASTEUR) Act, first introduced in 2020, and was most recently updated and reintroduced in the US Congress in 2026.^32^ The act proposes setting up a Committee on Critical Need Antibacterials, to award subscription-based contracts to developers of new antibacterials that address unmet medical need and provide substantial clinical value. ^32^ To qualify for a contract, the antibacterial must be designated ‘critical need’. It is envisaged a committee will develop a list of prioritised infections requiring novel antibacterials, considering unmet clinical need and an assessment of antibacterial resistance threats in the US by the Centers for Disease Control and Prevention (CDC). ^32^

Proposed product evaluation criteria to determine what payments contract holders receive include: improved clinical outcomes for patients with MDR infections; dose frequency; level of toxicity; reduction in adverse events, and the routine of administration. Innovative characteristics of the antibacterial will be assessed including addressing unmet medical needs for treatment of serious or life-threatening infections; membership of a new chemical class or novel target or mode of action, and containing no active ingredient already licensed. Benefits to the US health system and public health will also be examined including not being affected by cross-resistance to existing antibacterials; manufacturing capabilities in the US; product stability and storage; increased activity again resistance mechanisms; and reduction of the economic or population burden of AMR in the US.^32^ Proposed company criteria include its ability to ensure commercial availability within 30 days of contract initiation; maintain a reliable supply chain; conduct post-marketing studies; and implement stewardship and education strategies.^32^

2.1.3. *Canada*

In the Government of Canada’s 2023 budget, Canada committed to develop a pilot project to secure access to new antibacterials. Canada's Antibacterial Economic Incentives Pilot Project was intended to be a subscription model for FY 2024/25. Detailed information on eligibility and evaluation criteria were not available during our searches.^33^ In 2023, Public Health Agency of Canada (PHAC) worked with clinicians and pharmacists to identify antibacterials of interest and priority pathogens for the pilot project (Table 3). All five pathogens are on the WHO priority pathogens list and on Health Canada’s Pathogens of Interest List.

A 2023 report from the Council of Canadian Academies outlined potential product eligibility and evaluation criteria used elsewhere, including target pathogens, clinical value, public health needs, effectiveness, resistance profiles, and health system benefits.^34^ The report also discussed the importance of ensuring compliance with access, stewardship, and reporting requirements.

2.1.4. *European Union*

The EU’s Health Emergency Preparedness and Response Authority (DG-HERA) has worked on developing an EU-wide pilot revenue guarantee scheme for antibacterials.^35^ A contract note was issued in 2024 for a programme with pharmaceutical company Merini – ‘Pull incentive pilot for the procurement of availability and accessibility services of meropenem/vaborbactam (Vaborem ®) antibacterial in the European Union’^36^ – which was subsequently cancelled.^37^ We were unable to identify eligibility and evaluation criteria which determined Vaborem® as the eligible antibacterial, or Merini as the eligible company.

A political agreement was made in December 2025 to revise EU pharmaceutical legislation, to include a voluntary revenue guarantee scheme for joint procurement of antibacterials and the use of transferable exclusivity extensions (TEEs) to be granted to market authorisation holders of ‘priority antibacterials’.^38^ The details on eligibility and evaluation criteria for the voluntary revenue guarantee are yet to be developed, with plans for the European Commission in consultation with Member states, to draw up guidance on the methodology for valuing and remunerating products.

TEEs can be used to extend the market exclusivity period of any product in the EU region for one year, and can be purchased by any other pharmaceutical company. ^38^ The proposed revisions also include a ‘blockbuster clause’, which stipulates that the TEE cannot be used on products with annual gross sales of more than €490 million in the preceding four years. The political agreement is now subject to formal approval by the European Parliament and the Council.

A ‘priority’ antibacterial must meet at least one of the following criteria, as evaluated by the EMA: its mechanism of action differs significantly from any existing EU-authorised antibacterial; or it contains a new active substance that when used either alone or in combination with other active substances addresses a serious or life threatening infection. ^38^ In assessment of these criteria, the EMA will also take into account the WHO priority pathogen list, or an equivalent list established at the EU level (although not yet established).

The applicant for a TEE voucher must demonstrate the ability to supply the antibacterial in sufficient quantities to meet EU market needs; submit for regulatory approval to the EMA no later than 180 days after submission outside the EU; make publicly available sources and amounts of funding received for the antibacterial's R&D. ^38^

2.1.5. *Australia*

Australia’s independent Health Technology Assessment (HTA) Policy and Methods Review Reference Committee made recommendations in 2024 for funding mechanisms and economic models for antibacterial innovation and access.^39^ These included exempting new antibacterials that target WHO-listed priority pathogens from HTA fee requirements, and developing a framework to update HTA antibacterial policy. The committee advised exploring pull incentive mechanisms, e.g. price and volume delinking, advanced market commitments, and supply guarantees. It recommended the implementation and evaluation of a pilot subscription fund for novel antibacterials similar to the UK subscription model.

2.1.6. *Switzerland*

In Switzerland, there are proposed revisions to the Swiss Epidemics Act to allow a subscription fund model to incentivise antibacterial innovation and access.^40,41^ The independent Swiss Round Table on Antibiotics has undertaken work to identify the most critical gaps in effectiveness of antibacterials internationally and in Switzerland, and to examine the suitability of different pull incentive mechanisms for that context.^42^

# References

1	So AD, Shah TA. New business models for antibiotic innovation. Ups J Med Sci 2014; 119: 176–80.

2	Payne DJ, Miller LF, Findlay D, Anderson J, Marks L. Time for a change: addressing R&D and commercialization challenges for antibacterials. Philos Trans R Soc B Biol Sci 2015; 370: 20140086.

3	Årdal C, Outterson K, Hoffman SJ, et al. International cooperation to improve access to and sustain effectiveness of antimicrobials. Lancet Lond Engl 2016; 387: 296–307.

4	Kostyanev T, Bonten MJM, O’Brien S, et al. The Innovative Medicines Initiative’s New Drugs for Bad Bugs programme: European public-private partnerships for the development of new strategies to tackle antibiotic resistance. J Antimicrob Chemother 2016; 71: 290–5.

5	Laxminarayan R, Impalli I, Rangarajan R, et al. Expanding antibiotic, vaccine, and diagnostics development and access to tackle antimicrobial resistance. The Lancet 2024; 403: 2534–50.

6	McClelland S, Lamoureux B, Larson E. Trends in antimicrobial resistance legislation 2011-2019: A review of the US policy response to the antimicrobial resistance threat and its public health Impact. Am J Infect Control 2021; 49: 813–7.

7	Ohashi T, Nagashima M, Kawai N, Ohmagari N, Tateda K. A narrative review on drug development for the management of antimicrobial- resistant infection crisis in Japan: the past, present, and future. Expert Rev Anti Infect Ther 2022; 20: 1603–14.

8	Outterson K, Rex JH. Evaluating for-profit public benefit corporations as an additional structure for antibiotic development and commercialization. Transl Res J Lab Clin Med 2020; 220: 182–90.

9	Piddock LJV, Alimi Y, Anderson J, et al. Advancing global antibiotic research, development and access. Nat Med 2024; 30: 2432–43.

10	Renwick MJ, Brogan DM, Mossialos E. A systematic review and critical assessment of incentive strategies for discovery and development of novel antibiotics. J Antibiot (Tokyo) 2016; 69: 73–88.

11	Sciarretta K, Røttingen J-A, Opalska A, Van Hengel AJ, Larsen J. Economic Incentives for Antibacterial Drug Development: Literature Review and Considerations From the Transatlantic Task Force on Antimicrobial Resistance. Clin Infect Dis Off Publ Infect Dis Soc Am 2016; 63: 1470–4.

12	Simpkin VL, Renwick MJ, Kelly R, Mossialos E. Incentivising innovation in antibiotic drug discovery and development: progress, challenges and next steps. J Antibiot (Tokyo) 2017; 70: 1087.

13	Singer AC, Kirchhelle C, Roberts AP. (Inter)nationalising the antibiotic research and development pipeline. Lancet Infect Dis 2020; 20: e54–62.

14	Sinha MS, Kesselheim AS. Regulatory Incentives for Antibiotic Drug Development: A Review of Recent Proposals. Bioorg Med Chem 2016; 24: 6446–51.

15	Vogler S, Habimana K, Haasis MA, Fischer S. Pricing, Procurement and Reimbursement Policies for Incentivizing Market Entry of Novel Antibiotics and Diagnostics: Learnings from 10 Countries Globally. Appl Health Econ Health Policy 2024; 22: 629–52.

16	Anderson M, Panteli D, Mossialos E. How can the EU support sustainable innovation and access to effective antibiotics? Policy options for existing and new medicines. Copenhagen (Denmark): European Observatory on Health Systems and Policies, 2023 http://www.ncbi.nlm.nih.gov/books/NBK594073/ (accessed April 14, 2026).

17	Anderson M, Panteli D, van Kessel R, Ljungqvist G, Colombo F, Mossialos E. Challenges and opportunities for incentivising antibiotic research and development in Europe. Lancet Reg Health Eur 2023; 33: 100705.

18	Gotham D, Moja L, van der Heijden M, Paulin S, Smith I, Beyer P. Reimbursement models to tackle market failures for antimicrobials: Approaches taken in France, Germany, Sweden, the United Kingdom, and the United States. Health Policy Amst Neth 2021; 125: 296–306.

19	Renwick MJ, Simpkin V, Mossialos E. Targeting innovation in antibiotic drug discovery and development. 2016. http://www.lse.ac.uk/LSEHealthAndSocialCare/pdf/Antibiotics-book-web.pdf (accessed April 14, 2026).

20	Renwick M, Brogan D, Mossialos E. A Critical Assessment of Incentive Strategies for Development of Novel Antibiotics. 2014. https://amr-review.org/sites/default/files/ESRC%20ABX%20Incentives%20Review.pdf (accessed April 14, 2026).

21	Kelly R, Zoubiane G, Walsh D, Ward R, Goossens H. Public funding for research on antibacterial resistance in the JPIAMR countries, the European Commission, and related European Union agencies: a systematic observational analysis. Lancet Infect Dis 2016; 16: 431.

22	Årdal CO, Findlay D, Savic M, et al. Revitalizing the antibiotic pipeline: Stimulating innovation while driving sustainable use and global access. DRIVE-AB 2018. https://drive-ab.eu/wp-content/uploads/2018/01/CHHJ5467-Drive-AB-Main-Report-180319-WEB.pdf (accessed April 14, 2026).

23	Årdal C, Røttingen J-A, Opalska A, Van Hengel AJ, Larsen J. Pull Incentives for Antibacterial Drug Development: An Analysis by the Transatlantic Task Force on Antimicrobial Resistance. Clin Infect Dis Off Publ Infect Dis Soc Am 2017; 65: 1378–82.

24	Dutescu IA, Hillier SA. Encouraging the Development of New Antibiotics: Are Financial Incentives the Right Way Forward? A Systematic Review and Case Study. Infect Drug Resist 2021; published online Feb 5. https://www.tandfonline.com/doi/abs/10.2147/IDR.S287792 (accessed April 14, 2026).

25	Bhavnani SM, Krause KM, Ambrose PG. A Broken Antibiotic Market: Review of Strategies to Incentivize Drug Development. Open Forum Infect Dis 2020; 7: ofaa083.

26	Savic M, Årdal C. A Grant Framework as a Push Incentive to Stimulate Research and Development of New Antibiotics. J Law Med Ethics 2018; 46: 9–24.

27	Wasan H, Singh D, Reeta KH, Gupta YK. Landscape of Push Funding in Antibiotic Research: Current Status and Way Forward. Biology 2023; 12: 101.

28	Cama J, Leszczynski R, Tang PK, et al. To Push or To Pull? In a Post-COVID World, Supporting and Incentivizing Antimicrobial Drug Development Must Become a Governmental Priority. ACS Infect Dis 2021; 7: 2029–42.

29	Mattar C, Edwards S, Baraldi E, Hood J. An overview of the global antimicrobial resistance research and development hub and the current landscape. Curr Opin Microbiol 2020; 57: 56–61.

30	Ministry of Health, Labour and Welfare. Materials from the 1st Meeting of the Study Group on Market Incentives for Antimicrobials. 2023. https://www.mhlw.go.jp/stf/newpage_32303.html (accessed April 14, 2026).

31	Rep. Davis DK [D-I-7. Text - H.R.4127 - 117th Congress (2021-2022): DISARM Act of 2021. 2021; published online June 25. https://www.congress.gov/index.php/bill/117th-congress/house-bill/4127/text (accessed April 14, 2026).

32	Carter EL ‘Buddy’ [R-G-1. H.R.7352 - 119th Congress (2025-2026): PASTEUR Act of 2026. 2026; published online April 2. https://www.congress.gov/bill/119th-congress/house-bill/7352/text (accessed April 14, 2026).

33	Canada PHA of. Pilot project to make more antimicrobials available in Canada. 2024; published online Sept 17. https://www.canada.ca/en/public-health/services/antimicrobial-resistance/what-canadas-doing/pilot-project-make-more-antimicrobials-available.html (accessed April 14, 2026).

34	CCA. Overcoming Resistance: Expert panel on Antimicrobial Availability. 2023. https://cca-reports.ca/reports/pull-incentives-for-high-value-antimicrobials/ (accessed April 14, 2026).

35	European Commission. Meeting of the HERA Board. 2024. https://health.ec.europa.eu/document/download/ec6b2b06-2460-4e3c-a01e-97a94b0ee6eb_en?filename=hera_20240116_mi_en.pdf (accessed April 14, 2026).

36	EU Tenders: Pull incentive pilot for the procurement of availability and accessibility services of Vaborem antibiotic in the European Union. 2024. https://ted.europa.eu/en/notice/-/detail/741620-2024 (accessed April 14, 2026).

37	EU Tenders: Research and development services and related consultancy services cancellation notice for 9399-2025. 2025. https://ted.europa.eu/en/notice/-/detail/9399-2025 (accessed April 14, 2026).

38	Council of the European Union. Regulation laying down Union procedures for the authorisation and supervision of medicinal products for human use and establishing rulesgoverning the European Medicines Agency - Analysis of the final compromise text with a view to agreement. 2026. https://data.consilium.europa.eu/doc/document/ST-6366-2026-INIT/en/pdf (accessed April 14, 2026).

39	Accelerating Access to the Best Medicine for Australians Now and into the Future. 2024. https://www.health.gov.au/sites/default/files/2024-09/health-technology-assessment-policy-and-methods-review-final-report_0.pdf (accessed April 14, 2026).

40	Swiss Rountable on Antibiotics. RTA Newsletter December 2025. 2025. https://roundtableantibiotics.ch/en/news/rta-newsletter-december-2025 (accessed March 30, 2026).

41	Swiss Federal Authorities. Report on the results of the consultation relative to the partial revision of the Epidemic Act. 2025. https://www.fedlex.admin.ch/filestore/fedlex.data.admin.ch/eli/dl/proj/2023/50/cons_1/doc_18/fr/pdf-a/fedlex-data-admin-ch-eli-dl-proj-2023-50-cons_1-doc_18-fr-pdf-a.pdf (accessed March 30, 2026).

42	Swiss Rountable on Antibiotics. Effective antibiotics for the Swiss health care system: today and in the future. 2024. https://boris.unibe.ch/194363/1/2024%20RTA%20-%20Effective%20antibiotics%20for%20the%20Swiss%20health%20care%20system.pdf (accessed April 14, 2026).
